# Routine In-Hospital Radiographs Following Anterior Cervical Discectomy and Fusion Surgery: Neither Necessary nor Cost-Effective?

**DOI:** 10.7759/cureus.19975

**Published:** 2021-11-28

**Authors:** Rory M McCabe, Melvin Grainger, James Davis

**Affiliations:** 1 Trauma and Orthopaedics, Musgrove Park Hospital, Taunton, GBR; 2 Neurosurgery, Queen Elizabeth Hospital Birmingham, Birmingham, GBR

**Keywords:** complication, orthopaedics, neurosurgery, post-operative x-rays, post-operative radiographs, spinal surgery, anterior cervical discectomy and fusion, acdf

## Abstract

Background

Despite a paucity of evidence or literature to support routine in-hospital post-operative radiographs (POXR) of anterior cervical discectomy and fusion (ACDF) surgery, it remains accepted practice. Most spinal surgeons consider it part of their standard post-operative routine for ACDF despite nearly always documenting a ’satisfactory intra-operative image’ at the end of the operation. With an increasing financial pressure on NHS resources, our investigations should be clinically justified and evidence-based.

Purpose

To evaluate whether a post-operative radiograph of the cervical spine before discharge is either clinically justified or cost-effective in patients who have undergone an ACDF, despite having satisfactory intra-operative imaging.

Design

A retrospective review of 101 consecutive ACDF patients of radiographs performed before discharge, associated length of inpatient stay, and any complications involved.

Methods

A retrospective review was performed of 101 ACDF patients who had single or multi-level instrumentation for degenerative spinal disease from a single neurosurgical centre from all surgeons. Seventy-eight had an in-hospital post-operative anteroposterior (AP) and lateral radiograph, 23 did not. In 95 of these, it was documented that there was ‘satisfactory intra-operative imaging’ before the closure of skin, six lacked documentation of this. All patients had intra-operative imaging of completed instrumentation on the radiology system. Any post-operative complications were noted, and the length of hospital stay (LOS) was recorded. Six patients underwent ACDF following trauma, therefore leaving 95 elective cases. Study parameters also included: number of levels operated on, whether or not a plate was used with a cage, hospital costings for 2-view imaging and additional days of inpatient stay.

Results

There was one out of our 101 patients where the post-operative radiograph confirmed unsatisfactory placement of metalwork and warranted a return to surgery. However, the decision to perform this x-ray was based purely on the deteriorating post-operative clinical picture. In the cohort that had POXR’s, the average length of stay was 66.7 hours. Without POXR, it was 21 hours. The additional cost to the trust of performing the in-hospital radiographs was calculated to be £71,523 per year.

Conclusion

In patients who undergo ACDF surgery with an uneventful post-operative course and have satisfactory intra-operative imaging, in-hospital post-operative radiographs serve no clinical purpose and delay discharge. This gives additional cost to the trust, unnecessary radiation exposure and occupies potential bedspace.

## Introduction

Anterior cervical discectomy and fusion (ACDF) is a common operation performed for degenerate cervical disc disease, offered when one or more levels is causing symptoms of myelopathy or radiculopathy. A large proportion of spinal fusion procedures utilise instrumentation. A vital part of the operation is confirming the level at which you are operating following the exposure. This is done with intra-operative fluoroscopy. It is then widely the practice to repeat an intra-operative image once the instrumentation has been performed to again check document its level and correct position implant. In addition to this, it is common for a formal anteroposterior (AP) and lateral radiograph to be performed before being discharged, normally post-op day one or two. This is despite a paucity of evidence to support their use. In an NHS that has increasing financial pressures, we should have clinical justification for every investigation ordered [1]. There should also be clinical justification for radiation exposure.

Many studies in the literature [2,3] have examined the utility and cost of repeated post-operative radiographs, particularly in uncomplicated patients, particularly in joint replacement. They have found, in several, to have no use and have led to a change in practice. For example, it is now many clinicians practice to not perform radiographs after a hip hemiarthroplasty [4].

With the aim of improving care to our patients and streamlining their surgical journey, we evaluated the steps within our usual treatment of ACDF patients. The requirement of obtaining post-operative radiographs appears to have no recommendation within the literature. There are several papers from Europe and the US, finding them to be of no use [3,5-7]. Therefore, we performed this study to evaluate whether post-operative plain radiographs influenced the medical or surgical management of these patients before discharge and if performing them had an impact on the length of hospital stay.

## Materials and methods

The imaging was performed on all patients that underwent ACDF surgery in our neurosurgical centre (Department of neurosurgery, Queen Elizabeth Hospital, Birmingham) between close to a 12-month period (22.1.19 to 26.11.19) was retrospectively evaluated (n=101). Exclusion criteria were patients who received ACDF surgery due to trauma. These patients would often not have radiographs post-operatively, instead often CT scans, but also had a far greater length of hospital stay owing to their multitude of other injuries. This left 95 patients, of which all underwent intra-operative fluoroscopy - Seventy-eight of those received anteroposterior and lateral radiographs before discharge. Twenty-three had no post-operative radiographs (POXR) due to surgeon preference.

In addition to this, data was collected on indication for surgery, levels operated on, length of inpatient stay, whether a ‘stand-alone cage’ was used or ‘cage and plate’, satisfactory intra-operative imaging documented on the operation note, any post-operative complications encountered, and any need for return to theatre. This was done by review of medical records and imaging for the inpatient admission.

The additional cost to the hospital was then calculated per year of performing post-operative radiographs on ACDF surgeries. Included was the cost of an overnight bed as well as the cost of two radiographs. Additional cost which was not able to be included would have also been the wages of the porters and radiographers involved.

A literature review was conducted of previous studies that were looking at similar subjects. Pubmed and Medline were used for searches. Search terms included anterior cervical discectomy and fusion, post-operative radiograph. 

## Results

Demographics

101 consecutive ACDF patients from our neurosurgical centre were reviewed. 78 patients had post-operative AP and lateral radiographs before discharge. 23 patients had no radiographs performed and were discharged. 93% of this patient cohort had clear documentation of ‘satisfactory intra-operative imaging’ using fluoroscopy to confirm the positioning of the instrumentation at the end of the case, normally performed just prior to closure of skin.

Age distribution was between a range of 28 to 80. Average age of 50.8.

Number of levels

The distribution of discectomies was examined. All operations were between the levels of C3/4 and C6/7 intervertebral discs. A total of 142 levels operated on in the 101 surgeries. On average, 1.43 levels were operated on (mean), mode = 1, median = 1, with the most common level being C5/6. See Tables 1 and 2.

**Table 1:** Number of levels operated on per case

Number of levels operated on per case	No. of cases
1 (level)	68
2	25
3	7
4	1
Total number of cases	101
Total number of levels	143
Average levels per case	1.42

**Table 2:** Distribution of levels

Level	No.
C3/4	23
C4/5	31
C5/6	56
C6/7	33
Total	143

Hospital stay

The length of hospital stay was compared between the group who underwent post-operative radiographs (n=75) and the group who did not (n=20) following the removal of trauma patients. 

In the group that underwent POXR, the average length of hospital stay (LOS) was 66.5 hours (2.77 days). In the no POXR group, the average LOS was 21 hours (0.87 days).

Complications

Within the 95 cases, only four post-operative complications were observed. These were urinary retention, pseudomeningocoele, vertebral artery pseudoaneurysm, and loose metalwork (returned to theatre and re-sited). The first three are potential operative complications and risks, the final one is related to the prosthesis. In this particular case, the decision to perform a radiograph of the c-spine was taken post-operatively due to the patient complaining of severe pain and new neurology. The screws were noted to be slightly loose and had backed out. The decision was taken to return the patient to theatre and re-site the screws. 

Cost

The additional financial burden to the hospital of performing these POXRs was calculated. This was done by multiplying the average LOS of the POXR patients with the cost of a bed for 24 hours in our centre on our typical ward (£298), then adding the cost of two radiographs per patient (£73). Deducted from this was the average cost of a hospital stay of a patient who had no POXR. This difference was then multiplied by the average number of ACDF surgeries per year in our centre to produce a total of £71,523 per year. See Tables 3 and 4 below. Note: calculations do not include further costs of porters required for transporting patients, radiographers to perform X-ray (XR), and nursing staff on ward, which are difficult to quantify. 

**Table 3:** Cost of cervical XRs and overnight stay in bed XR - X-ray, AP - anteroposterior, LAT - lateral

Cost of cervical XR	(single XR) £36.79	(AP+LAT) £73.58
Cost of overnight stay		£298
Total		£371.58

**Table 4:** Calculations of additional cost caused to trust by performing POXRs LOS - length of hospital stay, POXR - post-operative radiographs

LOS (without trauma)	Days	Hours	Cost	/year
LOS - POXR	2.77	66.51	£899.35	£100,727.19
LOS - no POXR	0.88	21	£260.75	£29,204.00
				£71,523.19 (total cost)

## Discussion

Despite there being an abundance of literature defining the outcomes of degenerative spinal fusions, there is almost none supporting the clinical value of routine POXR following ACDF surgery [8,6]. Many spinal surgeons would consider AP and lateral radiographs to be an essential part of their post-operative management of instrumented spinal fusion surgery, despite previously documenting that there was satisfactory intra-operative fluoroscopy at the end of the case.

We have shown that patients’ clinical post-operative course and management have not been altered by the use of POXR. One single complication was encountered in the cohort, which was related to the implant; however, the decision was made in this case to perform POXRs due to the clinical picture of the patient in the immediate post-operative period. In this patient, they presented with new neurology and pain following surgery and was found to have loosening of the screws, which were re-sited in theatre. This patient was not planned to have a POXR; however, the decision changed once the clinical picture deteriorated. 

We also did a literature review of previous similar studies to this one (Table 5). All studies concluded that there was a 0% change in treatment related to performing the POXR. There was also a less than 6% complication rate in all studies. 

**Table 5:** Literature review of previous similar studies

Paper	Patients	Age (mean)	Levels (median)	Complications	Post-op X-ray	Change in treatment due to X-ray
Bartels et al. [5]	132	49.3	1	0%	97.0%	0%
Shau et al. [7]	103	51.8	2	None reported	76.7%	0%
Molinari et al. [6]	30	40.9	1	None reported	100%	0%
Ugokwe et al. [9]	53	57.7	1	5.7%	100%	0%
Martin et al. [10] - group 1 (had post-op. X-ray)	109	51.1	1	4.6%	96.3%	0%
Martin et al. [10] - post-op x-ray if indicated	113	52.1	1	6.2%	19.5%	0%
Current study	95	50.8	1	5.9%	82.1%	0%
Total	635	50.5	1	2.5%	84.2%	0%

We are therefore not endorsing the cessation of all POXRs in ACDF but rather that they should only be performed when clinically indicated. The results of this study show that satisfactory intra-operative fluoroscopy is sufficient to demonstrate correct placement of instrumentation in 100% of the patients who went on to have an uncomplicated post-operative course. It is also standard practice to perform radiographs on the first outpatient visit, and so should a clinician be concerned about migrating or movement of metalwork, this would be a better time to pick this up and has no effect on inpatient bedspace. Other reasons suggested for wanting radiographs are for medico-legal documentation or for surgical trainee teaching, for both of which an outpatient X-ray should be sufficient, which in turn would avoid the extended stay as an inpatient. 

A survey in 1998 of 118 consultant neurosurgeons’ routine practice surrounding ACDF surgery in the UK and Ireland found that 80% perform routine post-operative radiographs [11]. Forty per cent said they would also perform further imaging at outpatient follow-up. A more up to date survey of current practices of UK consultants would be interesting to compare to the results of this study.

A retrospective and prospective study by Bartels et al. in 2009 [5] looked at the levels of subsidence of the cage at different intervals from the operation. It showed that, at day one, 0% of cases had subsided; however, at six weeks, 14.5% had subsided. This finding, however, never changed management or prompted intervention so long as the patient did not have any complaints.

This study was limited to the fact it was a retrospective study of a patient cohort over a one year time period. Patients were not randomised to have either XR or no XR. Martin et al. [10] did this in their study, which had similar findings and conclusions. It was limited to one neurosurgical centre in the UK; the power of the results would be strengthened by analysing the practice of multiple centres performing ACDF surgery. 

## Conclusions

Our study combined with a review of previous literature shows no clinical reason to support routine post-operative radiographs following ACDF surgery. They do not influence patient care and delay discharge, inferring additional cost to the health service and slowing of inpatient bed flow. They do not result in a lower rate of complications, need for further imaging or further surgery. In the process, we are exposing patients to unnecessary ionising radiation with no clinical indication.

We suggest that the practice of obtaining routine radiographs of the cervical spine should be abandoned unless there is a clear clinical indication.
